# Mild hypoglycemia is independently associated with increased mortality in the critically ill

**DOI:** 10.1186/cc10322

**Published:** 2011-07-25

**Authors:** James S Krinsley, Marcus J Schultz, Peter E Spronk, Robin E Harmsen, Floris van Braam Houckgeest, Johannes P van der Sluijs, Christian Mélot, Jean Charles Preiser

**Affiliations:** 1 Division of Critical Care, Stamford Hospital, Columbia University College of Physicians and Surgeons, 190 West Broad Street, Stamford, CT 06902, USA; 2 Department of Intensive Care, Academic Medical Center, University of Amsterdam, Meibergdreef 9, 1105 AZ Amsterdam, The Netherlands; 3 Laboratory of Experimental Intensive Care and Anesthesiology, Academic Medical Center, University of Amsterdam, Meibergdreef 9, 1105 AZ Amsterdam, The Netherlands; 4 Department of Intensive Care, Gelre Hospitals, Lukas, Albert Schweitzerlaan 31, 7334 DZ Apeldoorn, The Netherlands; 5 Department of Intensive Care, Tergooi Hospitals, Blaricum, Rijksstraatweg 1, 1261 AN Blaricum, The Netherlands; 6 Department of Intensive Care Medicine, Medical Center Haaglanden, Lijnbaan 32, The Hague, 2512 VA Den Haag, The Netherlands; 7 Department of Emergency Medicine, Erasme University Hospital, Route de Lennik 808, 1070 Bruxelles, Belgium; 8 Department of Intensive Care, Erasme University Hospital, Route de Lennik 808, 1070 Bruxelles, Belgium

## Abstract

**Introduction:**

Severe hypoglycemia (blood glucose concentration (BG) < 40 mg/dL) is independently associated with an increased risk of mortality in critically ill patients. The association of milder hypoglycemia (BG < 70 mg/dL) with mortality is less clear.

**Methods:**

Prospectively collected data from two observational cohorts in the USA and in The Netherlands, and from the prospective GLUCONTROL trial were analyzed. Hospital mortality was the primary endpoint.

**Results:**

We analyzed data from 6,240 patients: 3,263 admitted to Stamford Hospital (ST), 2,063 admitted to three institutions in The Netherlands (NL) and 914 who participated in the GLUCONTROL trial (GL). The percentage of patients with hypoglycemia varied from 18% to 65% among the different cohorts. Patients with hypoglycemia experienced higher mortality than did those without hypoglycemia even after stratification by severity of illness, diagnostic category, diabetic status, mean BG during intensive care unit (ICU) admission and coefficient of variation (CV) as a reflection of glycemic variability. The relative risk (RR, 95% confidence interval) of mortality associated with minimum BG < 40, 40 to 54 and 55 to 69 mg/dL compared to patients with minimum BG 80 to 109 mg/dL was 3.55 (3.02 to 4.17), 2.70 (2.31 to 3.14) and 2.18 (1.87 to 2.53), respectively (all *P *< 0.0001). The RR of mortality associated with any hypoglycemia < 70 mg/dL was 3.28 (2.78 to 3.87) (*P *< 0.0001), 1.30 (1.12 to 1.50) (*P *= 0.0005) and 2.11 (1.62 to 2.74) (*P *< 0.0001) for the ST, NL and GL cohorts, respectively. Multivariate regression analysis demonstrated that minimum BG < 70 mg/dL, 40 to 69 mg/dL and < 40 mg/dL were independently associated with increased risk of mortality for the entire cohort of 6,240 patients (odds ratio (OR) (95% confidence interval (CI)) 1.78 (1.39 to 2.27) *P *< 0.0001), 1.29 (1.11 to 1.51) *P *= 0.0011 and 1.87 (1.46 to 2.40) *P *< 0.0001) respectively.

**Conclusions:**

Mild hypoglycemia was associated with a significantly increased risk of mortality in an international cohort of critically ill patients. Efforts to reduce the occurrence of hypoglycemia in critically ill patients may reduce mortality

## Introduction

Hyperglycemia occurs commonly in critically ill patients and is strongly associated with increased risk of mortality [1-3]. Interventional trials designed to treat even moderate degrees of hyperglycemia with insulin have met with mixed success. A landmark single-center trial among a cohort of surgical intensive care unit (ICU) patients resulted in substantial reductions in mortality and morbidity in the treated patients [4]. Three years later a large observational study in a heterogeneous population of ICU patients reached a similar conclusion [5]. However, in the second Leuven trial of intensive insulin therapy (IIT), conducted in a cohort of medical patients, patients in the interventional arm did not demonstrate reduced mortality, but did have shorter ICU length of stay and duration of mechanical ventilation [6]. Subsequent trials of IIT were stopped prematurely due to high rates of hypoglycemia and protocol violations [7,8] or demonstrated higher mortality among the intensively treated cohort [9]. In fact, the second Leuven trial was the first investigation to identify an association between severe hypoglycemia (BG < 40 mg/dL) and mortality [6].

A relatively low rate of severe hypoglycemia, defined as blood glucose concentration (BG) < 40 mg/dL and identified as having an independent association with mortality in several large observational cohorts [10-12], was a key feature that distinguished the first two investigations [4,5] from those that followed [6-9]. The Leuven investigators subsequently pooled data from their surgical [4] and medical [6] trials and used multivariate analysis to confirm that severe hypoglycemia had a strong and independent association with mortality [13]. A recent large observational cohort study suggested that even mild levels of hypoglycemia, defined as BG < 72 mg/dL, were associated with increased risk of death among critically ill patients [14]. Consistently, the recently revised standards of medical care in diabetes used a threshold BG of 70 mg/dl to define hypoglycemia [15].

The purpose of this study was to evaluate the association of hypoglycemia, defined as BG < 70 mg/dL, with mortality. We hypothesized that (1) hypoglycemia (BG < 70 mg/dL) was associated with increased risk of mortality, and (2) that a glycemic management strategy that tolerated brief periods of mild hypoglycemia and corrected these events without creating marked glycemic excursions would demonstrate a weaker association with mortality than would protocols that avoided and treated mild hypoglycemia more vigorously. We had the unique opportunity to analyze a large and diverse group of critically ill patients in an international collaboration, including a large single-center cohort from 1 ICU in the United States of America, a multicenter cohort from 3 ICUs from The Netherlands, and 21 ICUs from Europe and Israel that participated in the GLUCONTROL trial, a multicenter randomized controlled trial of IIT [8].

## Materials and methods

### Settings

The adult ICU of Stamford Hospital (Stamford, CT, USA) is a 16-bed unit that treats a heterogeneous population of medical, surgical and trauma patients. Medical and surgical house staff, closely supervised by a team of intensivists, deliver care.

The adult ICUs of the three hospitals in The Netherlands (Gelre Hospital, Apeldoorn, The Netherlands; Tergooi Hospitals, Hilversum, The Netherlands; Medical Center Haaglanden, The Hague, The Netherlands) are a 10-bed, 9-bed and 18-bed unit, respectively, which treat a heterogeneous population of medical, surgical and trauma patients. A team of intensivists delivers care in a closed-format setting.

Twenty-one adult ICUs from 19 different hospitals in seven countries in Europe and Israel participated in the GLUCONTROL trial [8] The number of ICU beds of the participating units ranged from 5 to 44 (median 12).

### Patients

The patient cohort in Stamford (ST) included 3,263 patients admitted to the ICU between 12 January 2007 and 30 April 2010, who had at least three BG values obtained during their ICU stay. Forty-one patients admitted during this period with a diagnosis of diabetic ketoacidosis or hyperosmolar nonketotic coma were excluded from the present analysis.

The patient cohort in The Netherlands (NL) included 2,063 patients admitted to the three ICUs between 1 January 2007 and 29 December 2009, who had at least three BG values obtained during their ICU stay: 1,098 patients, admitted between 1 January 2007 and 31 January 2008 were subjected to a "loose" IIT guideline; 965 patients admitted between 1 February 2008 and 29 December 2009 were subjected to a "strict" IIT guideline (see below for details on "loose" and "strict" IIT). Per protocol, patients admitted during this period with a diagnosis of diabetic ketoacidosis or hyperosmolar nonketotic coma were not subjected to treatment according to the guidelines.

The patient cohort in the GLUCONTROL trial (GL) included 914 patients with at least three BG values; the other 164 patients (those with fewer than three BG recorded) were evenly distributed between the intensive (GL-IIT) and intermediate glucose control groups (GL-C).

### Glycemic control and blood glucose monitoring

The glycemic target in Stamford during the period of the investigation was 80 to 125 mg/dL. Details of the protocol have been published previously [16] (and are available as Additional file 1). Most of the BG measurements (85%) were made using bedside glucometers (Accu-Chek Inform, Indianapolis, IN, USA) and capillary or venous blood; the remainder were performed in the central laboratory using a Siemens Advia 1800 analyzer (Siemens Corporation US, Washington, DC, USA) or in the ICU using a GEM4000 point of care analyzer (Instrument Laboratory, Lexington, MA, USA).

Patients in the NL cohort were treated with loose or strict IIT. With loose glucose control, the three participating ICUs followed the 2004 Surviving Sepsis Campaign Guidelines [17], and aimed for a BG < 150 mg/dL. Insulin dose and route of administration (either intravenous or subcutaneous), and timing and type of BG measurement (either using capillary or arterial blood, at the bedside or in a central laboratory) were loosely defined in the guidelines in use. ICUs nurses practiced blood glucose control. With strict IIT, the ICUs followed an adjusted Leuven guideline with a glycemic target range of 80 to 110 mg/dL [18]; administration of insulin was intravenous at all times, and BG measurements were performed at the bedside. Notably, while the protocol aimed for BG > 80 mg/dL, the protocol allowed BG from 60 to 80 mg/dL if they were short in duration. In such case, insulin infusion was stopped. Small boluses of dextrose were given only in case of severe hypoglycemia. Blood glucose control required a high level of intuitive decision-making. All BG measurements were made using bedside glucometers (AccuChek Inform, Almere, The Netherlands) and arterial blood; capillary blood was never used.

In the GLUCONTROL trial, patients were randomized to IIT (target BG: 80 to 110 mg/dl) or intermediate glucose control (target BG 140 to 180 mg/dl), using an insulin protocol ([8], and available as Additional file 2). BG measurements were performed on arterial or central venous samples when a catheter was in place and a blood gas analyzer was used preferentially. Capillary samples and a specific glucometer (Accu to Check Inform, Roche Diagnostics, Mannheim, Germany) were allowed.

### Data collected

In Stamford, data were abstracted from the ICU's comprehensive clinical database, including prospective collection of admission diagnosis, demographic information and calculation of the Acute Physiology and Chronic Health Evaluation II (APACHE II) scores. The database is linked to the hospital's clinical information system to retrieve glucose values and the patient's hospital discharge status. Diabetic status was determined prospectively based on all available clinical information for every patient.

The Dutch centers' data were abstracted from the National Intensive Care Evaluation (NICE) database, maintained by the NICE Foundation [19], including prospective collection of admission diagnosis, demographic information and calculation of the APACHE II scores. The database was linked to the hospital's clinical information system to retrieve BG.

For the GL cohort, the data were abstracted from the original database recorded by the study participants. An independent biostatistician managed the web-based central database.

### Statistical analysis

Hypoglycemia was defined as BG < 70 mg/dL. Severe hypoglycemia was defined as BG < 40 mg/dL. Additional stratification included bands of a minimum BG of 40 to 54, 55 to 69, 70 to 79, 80 to 109 and ≥ 110 mg/dL We calculated the relative risk of mortality associated with increments of hypoglycemia compared to patients with a minimum BG of 80 to 109 mg/dL. Additional analyses stratified patients by mean BG during ICU stay using increments of 80 to 110 mg/dL, 110 to 140 mg/dL, 140 to 180 mg/dL and ≥ 180 mg/dL, as well as by severity of illness, using APACHE II scores, and by diabetic status and diagnostic category. We also calculated the coefficient of variation (CV) for each patient, defined as standard deviation of the mean BG/mean BG. Multivariate analysis to assess the independent association of hypoglycemia with mortality included the following parameters found to be statistically significant at *P *< 0.10 on univariate analysis: age, modified APACHE II score (age component deleted, in order to avoid colinearity with age in the multivariate analysis: age 45 to 54 two points; age 55 to 64 three points; age 65 to 74 five points; age ≥ 75 six points), ICU LOS, diagnostic category on admission to the ICU (medical vs. surgical), mechanical ventilation, mean BG and CV. Diabetes was not associated with mortality on univariate analysis and, therefore, was not entered into the multivariate model.

Continuous data were presented as median (interquartile range), and compared using the Mann Whitney rank sum test; all chosen parameters were not normally distributed. Ordinal data were presented as percentages and compared using the Chi square test. Mortality was defined throughout as hospital- (that is, not ICU-) mortality. Statistical analysis was performed using the MedCalc statistical package version 10.1.1.6.0 (MedCalc software, Broekstraat 52, 9030 Mariakerke, Belgium). A *P- *value of < 0.05 was considered statistically significant.

The Stamford Hospital institutional review board approved this investigation.

The GLUCONTROL trial was approved by each institutional review board of the 21 participating hospitals. Patients, or their designated surrogates, participating in the trial gave informed consent.

Approval to conduct the study was obtained from the medical ethics committees of the three centers of the Academic Medical Center in The Netherlands, which waived the requirement for individual patient-level consent.

## Results

### Cohort characteristics

Table 1 illustrates differences in clinical characteristics, outcomes and frequently used metrics of glycemic control among patients in the three cohorts, as well as aggregated data for the entire population of 6,240 patients. The percentage of patients with hypoglycemia varied considerably, as did mean glucose level, glycemic variability and mortality. Table 2 details the different outcomes of patients with and without hypoglycemia. For the entire cohort of 6,240, patients with hypoglycemia were older, had higher APACHE II scores, ICU LOS and mortality and also had higher indices of glycemic variability and lower mean BG during ICU stay (all comparisons *P *< 0.0001).

**Table 1:** Characteristics of the patient cohorts

	ALL	ST	NL-L	NL-S	GL-C	GL-IIT
**Demographics and outcomes**						
Number	6,240	3,263	1,098	965	460	454
Age	68 (54 to 78)	68 (53 to 80)	69 (57 to 78)	68 (56 to 78)	65 (53 to 74)	65 (51 to 74)
DM (%)	20.4	20.5	N/A	N/A	22.6	17.2
**Diagnostic category (%)**						
Medical	55.2	54.1	64.1	63.2	39.9	40.4
Surgical/Trauma	44.8	45.9	35.9	36.8	60.1	59.6
Mechanical ventilation (%)	45.8	35.4	N/A	N/A	84.2	82.0
ICU LOS	2.5 (1.1 to 5.7)	1.5 (0.9 to 3.1)	3.0 (1.8 to 6.9)	3.0 (1.7 to 6.3)	6.0 (3.0 to 13.0)	6.0 (3.0 to 12.5)
APACHE II score	16 (11 to 22)	14 (10 to 21)	19.7 (8.2)	19.4 (8.3)	15 (11 to 21)	15 (11 to 21)
Mortality (%)	19.2	14.2	27.5	26.0	17.8	22.9
**Glucose control parameters**						
Number of BG measurements per patient	18 (9 to 47)	15 (8 to 34)	17 (7 to 38)	29 (12 to 69)	29 (12 to 74)	37 (15 to 93)
Number of BG measurements per day*	8.7 (5.7 to 10.5)	9.3 (8.0 to 11.3)	5.1 (3.6 to 7.6)	9.8 (6.8 to 12.1)	4.5 (3.2 to 6.7)	5.6 (3.8 to 9.0)
Mean (mg/dL)	124.4 (112.2 to 140.7)	124.0 (112.0 to 138.0)	127.7 (116.4 to 145.0)	117.9 (107.0 to 137.0)	146.3 (128.1 to 164.6)	118.5 (109.3 to 130.3)
CV (%)	23.8 (16.7 to 32.6)	21.0 (14.8 to 28.5)	26.9 (18.8 to 35.2)	31.8 (23.8 to 40.8)	20.7 (15.4 to 26.2)	26.2 (20.3 to 33.1)
SD (mg/dL)	29.5 (20.0 to 42.6)	25.9 (17.7 to 37.5)	33.8 (23.6 to 49.4)	37.1 (27.3 to 50.3)	29.3 (19.9 to 41.7)	30.5 (22.7 to 42.1)
**Percentage of patients with hypoglycemia**						
< 40 mg/dL	6.7	2.9	8.8	18.1	2.4	9.5
40 to 54 mg/dL	12.6	8.4	13.6	25.2	3.9	23.1
55 to 69 mg/dL	17.7	14.9	19.3	21.6	11.5	31.3
< 70 mg/dL	37.0	26.2	41.7	64.9	17.8	63.9

APACHE II, Acute Physiology and Chronic Health Evaluation II disease classification system; BG, blood glucose; CV, coefficient of variation; DM, diabetes mellitus; GL-C, patients in the control arm of the GLUCONTROL trial; GL-IIT, patients in the intervention arm of the GLUCONTROL trial; LOS, length of stay (days); NL-L, Netherlands "loose" cohort; NL-S, Netherlands "strict" cohort; SD, standard deviation; ST, Stamford cohort.

*Calculated as (mean number of BG tests/mean ICU LOS). Data expressed as median (interquartile range), or percentages

**Table 2:** Characteristics of patients with hypoglycemia and patients without hypoglycemia

	ALL		ST		NL		GL	
	**HYPO**	**NON**	**HYPO**	**NON**	**HYPO**	**NON**	**HYPO**	**NON**

**Demographics and outcomes**								
Number	2,309	3,921	857	2,406	1,084	979	372	542
Age (years)	70 (57 to 79)	66 (52 to 71)	75 (60 to 83)	66 (51 to 78)	69 (58 to 78)	68 (55 to 78)	66 (51 to 75)	65 (52 to 73)
DM (%)	27.3	17.5	29.6	17.2	N/A	N/A	22.5	18.7
**Diagnostic Category (%)**								
Medical	64.5	49.8	69.7	48.5	65.5	61.7	48.7	33.4
Surgical/Trauma	35.5	50.2	30.3	51.5	34.5	38.3	51.3	66.6
Mechanical ventilation	63.3	38.5	55.0	28.5	N/A	N/A	82.6	83.5
ICU Length of Stay	5.0 (2.2 to 10.5)	1.8 (1.0 to 3.3)	3.0 (1.4 to 7.1)	1.2 (0.8 to 2.3)	5.2 (2.6 to 10.3)	2.0 (1.3 to 3.2)	9 (5 to 17)	5 (3 to 9)
APACHE II score	20 (14 to 26)	14 (10 to 19)	21.1 (9.3)	14.2 (8.1)	21.2 (7.8)	17.8 (8.4)	18.7 (7.5)	15.3 (6.5)
Mortality (%)	29.6	13.1	29.1	8.9^1 ^	30.1	23.2	29.6	14.0
**Glucose control parameters**								
Number of BG measurements per patient	45 (21 to 97)	11 (7 to 24)	35 (16 to 90)	11 (7 to 23)	47 (24 to 92)	9 (5 to 18)	69 (29 to 136)	20 (10 to 48)
Number of BG measurements per day*	9.5 (7.2 to 11.9)	8.0 (5.0 to 10.0)	11.7	9.2	9.0	4.5	7.7	4.0
Mean (mg/dL)^	118.3 (108.1 to 132.5)	128.1 (115.3 to 144.4)	120.3 (108.4 to 133.9)	125.4 (113.4 to 138.6)^1 ^	116.9 (107.5 to 129.9)	131.9 (119.2 to 152.6)	119.1 (109.0 to 136.3)	137.5 (121.0 to 158.4)
CV (%)	31.6 (25.0 to 40.0)	19.2 (13.7 to 26.1)	29.3 (23.1 to 38.2)	18.4 (13.0 to 24.6)	34.2 (23.0 to 42.4)	21.7 (15.0 to 30.2)	27.7 (22.6 to 35.5)	20.2 (15.2 to 25.8)
SD (mg/dL)	24.8 (16.7 to 36.0)	37.6 (28.9 to 50.4)	34.9 (26.2 to 48.8)	22.7 (15.4 to 32.5)	40.2 (31.2 to 54.1)	28.1 (18.8 to 44.5)	34.8 (25.7 to 46.6)	27.5 (19.1 to 38.8)

*Calculated as (mean number of BG tests/mean ICU LOS)

^These values represent the median (IQR) of the individual patients' mean BG

Data expressed as median (interquartile range), or percentages

APACHE II, Acute Physiology and Chronic Health Evaluation II disease classification system; BG, blood glucose; CV, coefficient of variation; DM, diabetes mellitus; GL, GLUCONTROL cohort; HYPO, hypoglycemia (BG < 70 mg/dL); LOS, length of stay (days); NL, Netherlands cohort; NON, no hypoglycemia; SD, standard deviation; ST, Stamford cohort.

### Sensitivity analysis: association between hypoglycemia and mortality

**Figure 1:**
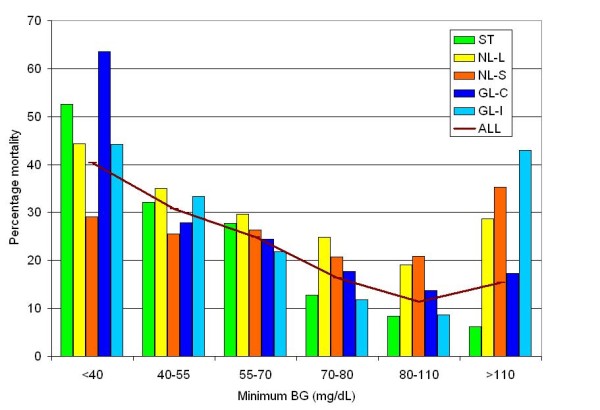
**Relationship between minimum BG during ICU stay and mortality, stratified by subpopulation**. BG, blood glucose; GL-C, GLUCONTROL-control arm; GL-I, GLUCONTROL-intensive arm; NL-L, Netherlands-"loose"; NL-S, Netherlands-"strict"; ST, Stamford.

**Figure 2:**
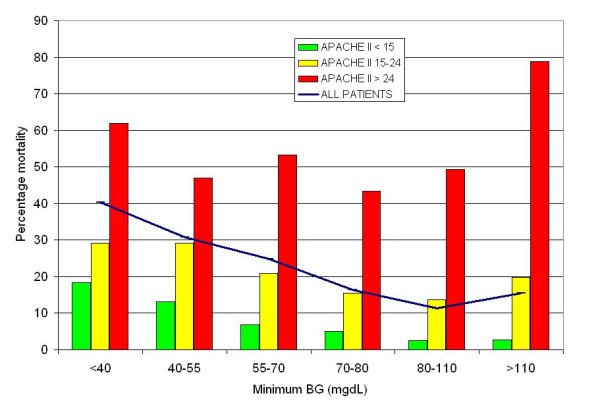
**Relationship between minimum BG during ICU stay and mortality, stratified by APACHE II score**. APACHE II, Acute Physiology and Chronic Health Evaluation II disease classification system; BG, blood glucose.

Table 3 demonstrates for the entire cohort of 6,240 patients increased relative risk (RR) of mortality for patients with minimum BG < 40, 40 to 54, 55 to 69, 70 to 79 compared to those with a minimum BG of 80 to 109 mg/dL and Figure 1 stratifies these results by subpopulation. Increasing severity of hypoglycemia was consistently associated with increased risk of mortality. The RR (95% CI) for mortality associated with hypoglycemia (BG < 70 mg/dL) in ST patients was 3.28 (2.78 to 3.87) *P *< 0.0001; for NL patients in the "loose" and "strict" cohorts it was 1.53 (1.27 to 1.86) *P *< 0.0001 and 1.10 (0.87 to 1.38) *P *= 0.4288, respectively; and for patients in the GL cohort it was 2.11 (1.62 to 2.74). Figure 2 stratifies this analysis by severity of illness. The association between mortality and mild hypoglycemia is strongly demonstrated for patients with a mild to moderate (APACHE II score < 15) and moderately severe (APACHE II score 15 to 24) disease but blunted for those with very severe disease (APACHE II score > 24).

**Table 3:** Relative risk of mortality: comparison to patients with a minimum BG of 80 to 110 mg/dL

Minimum BG (mg/dL)	Number of patients	RR (95% CI)	*P*-value
< 40	421	3.55 (3.02 to 4.17)	< 0.0001
40 to 55	789	2.70 (2.31 to 3.14)	< 0.0001
55 to 70	1,103	2.18 (1.87 to 2.53)	< 0.0001
70 to 80	854	1.43 (1.18 to 1.73)	0.0002
80 to 110	2,383	Reference	
≥ 110	690	1.35 (1.10 to 1.66)	0.0046

BG, blood glucose; CI, confidence interval; RR, relative risk

### Association between hypoglycemia and mortality, stratified by clinical characteristics of patients

Table 4 demonstrates that patients with hypoglycemia sustained higher mortality than did those without hypoglycemia, regardless of diagnostic category, diabetic status, ICU LOS and frequency of BG measurements. Table 5 displays the results of multivariate regression analysis of the association between hypoglycemia and mortality for patients with these clinical characteristics, as well as the independent association of mortality associated with BG < 70 mg/dL, BG 40 to 69 mg/dL and BG < 40 mg/dL.

**Table 4:** Mortality percentages of patients with hypoglycemia and without hypoglycemia, stratified by different clinical characteristics

Characteristic	Hypoglycemia	No hypoglycemia	*P*-value
**Diagnostic category**			
**Medical**	33.9	19.2	< 0.0001
**Surgical/trauma**	21.8	7.1	< 0.0001
**Diabetes**	27.2	10.5	< 0.0001
**No diabetes**	30.0	9.7	< 0.0001
**ICU LOS**			
**< 3 days**	28.3	11.4	< 0.0001
**3 to 7 days**	29.0	14.6	< 0.0001
**> 7 days**	30.6	22.2	0.0024
**Frequency of BG tests**			
**< 6/day**	25.2	14.4	< 0.0001
**6 to 9/day**	31.3	12.1	< 0.0001
**> 9/day**	29.9	13.0	< 0.0001

BG, blood glucose; LOS, length of stay

**Table 5:** Multivariate analysis

	Minimum BG < 40 mg/dL		Minimum BG 40 to 69 mg/dL		Minimum BG < 70 mg/dL	
	**OR (95% CI)**	***P*-value**	**OR (95% CI)**	***P*-value**	**OR (95% CI)**	***P*-value**

**Entire cohort^1 ^** **(*n *= 6,240)**	1.49 (1.14 to 1.94)	0.0031	1.17 (0.99 to 1.38)	0.0557	1.47 (1.22 to 1.78)	< 0.0001
**No diabetes^2 ^** **(*n *= 3,326)**	2.42 (1.42 to 4.10)	0.0011	1.33 (1.02 to 1.75)	0.0370	1.65 (1.24 to 2.19)	0.0005
**Diabetes^2 ^** **(*n *= 850)**	2.37 (1.12 to 5.01)	0.0236	1.58 (0.99 to 2.52)	0.0549	2.48 (1.48 to 4.15)	0.0005
**Medical admission^1 ^** **(*n *= 3,441)**	1.44 (1.02 to 2.03)	0.0395	1.06 (0.85 to 1.32)	0.6165	1.28 (1.00 to 1.63)	0.0472
**Surgical/trauma admission^1 ^(*n *= 2,787)**	1.59 (1.05 to 2.40)	0.0283	1.34 (1.04 to 1.72)	0.0220	1.77 (1.34 to 2.34)	< 0.0001

Multivariate model includes age; modified APACHE II score (age component deleted); CV; ICU LOS; mean BG; medical vs. surgical/trauma admission; mechanical ventilation (as indicated)

^1 ^Does not include mechanical ventilation in the model

^2 ^Excludes NL patients (for whom MV and diabetes data not available); includes MV in the model

APACHE II, Acute Physiology and Chronic Health Evaluation II disease classification system; BG, blood glucose; CV, coefficient of variation; LOS, length of stay; NL, Netherlands cohort; OR, odds ratio

### Mortality associated with number of hypoglycemic events

Among 4,906 patients with no or one episode of hypoglycemia, multivariate analysis demonstrated that a single episode of hypoglycemia was independently associated with increased risk of mortality: OR (95% CI) 1.31 (1.06 to 1.62) *P *= 0.0121. Among 5,535 patients with up to three episodes of hypoglycemia, multivariate analysis demonstrated that hypoglycemia was independently associated with increased risk of mortality: OR (95% CI) 1.35 (1.14 to 1.61) *P *= 0.0007 Among the entire cohort of 6,240 patients, patients with four or more episodes of hypoglycemia had OR for mortality: 1.49 (1.20 to 1.83) *P *= 0.0002 (compared to patients without hypoglycemia).

### Association between hypoglycemia and mortality, stratified by mean BG concentration and coefficient of variation

**Figure 3:**
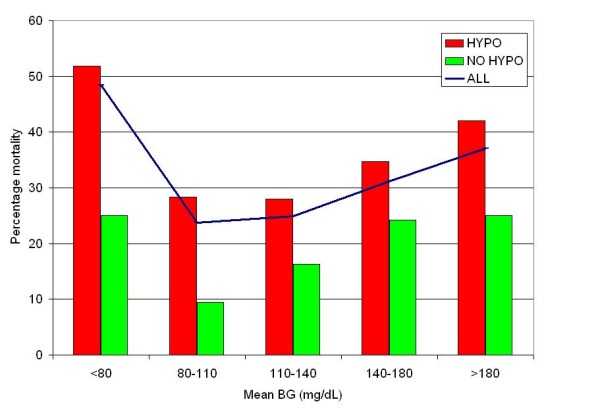
**Relationship of mean blood glucose-mortality, stratified by presence or absence of hypoglycemia**. There were 32 patients with mean BG < 80 mg/dL, 28 with hypoglycemia (51.9% mortality) and 1 without hypoglycemia (25% mortality). BG, blood glucose; HYPO; hypoglycemia BG < 70 mg/dL.

**Figure 4:**
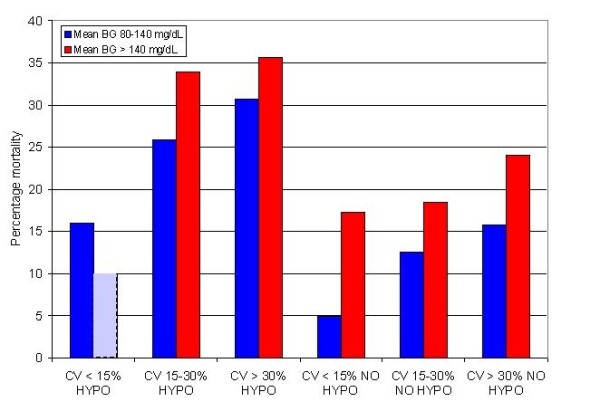
**Association of mortality with disturbances in the three domains of glycemic control**. There were no patients with Mean BG > 140 mg/dL and CV < 15%. BG, blood glucose; CV, coefficient of variation; HYPO, hypoglycemia BG < 70 mg/dL.

Figure 3 displays mortality stratified by bands of mean BG during ICU stay: < 80 mg/dL, 80 to 110 mg/dL, 110 to 140 mg/dL, 140 to 180 mg/dL and ≥ 180 mg/dL, in patients with and without hypoglycemia. Among patients with mean BG 80 to 110 mg/dL, hypoglycemia was associated with a 4-fold increase in mortality; the "hypoglycemia penalty" was 2.8-fold for patients with mean BG 110 to 140 and 2-fold for patients with a mean BG of 140 to 180 mg/dL. Among patients with CV < 15%, 15 to 30% and ≥ 30% mortality was higher in patients with hypoglycemia than in patients without hypoglycemia: 13.3% vs. 6.9% (*P *= 0.3216), 26.4% vs. 14.3% (*P *< 0.0001) and 32.5% vs. 21.2% (*P *< 0.0001). Finally, Figure 4 presents unadjusted mortality rates for patients with differing mean blood glucose, differing coefficient of variation of blood glucose and presence or absence of hypoglycemia. The highest crude mortality rates were observed in patients with higher mean blood glucose and coefficient of variation of blood glucose who experience hypoglycemia. Patients with hypoglycemia, high glycemic variability and high mean BG sustained 7.5-fold higher mortality than did those with low glycemic variability, low mean BG and no hypoglycemia.

## Discussion

This study examined the relationship between hypoglycemia occurring during ICU stay and hospital mortality in three cohorts of patients. The salient finding is that even a single episode of mild hypoglycemia, defined as BG < 70 mg/dL, was associated with increased risk of mortality. A major strength of this investigation includes the nature of the aggregated patient cohort, involving patients from different countries, with varying severities of illness and ICU LOS, treated in ICU's using different glycemic targets, measurement technologies and glycemic management protocols. Notably, the association between hypoglycemia and mortality was different among cohorts with different strategies of glucose control. The highest relative risk for mortality was seen in the cohort with the lowest rates of hypoglycemia while the lowest risk for mortality was seen in the cohort in whom short episodes of mild hypoglycemia were accepted as part of the guideline for IIT. The association between hypoglycemia and mortality was independent of diagnostic category and diabetic status and was seen predominantly in patients with mild to moderate and moderate to severe disease on presentation to the ICU, reflected by APACHE II scores 0 to 14 and 15 to 24 respectively. Finally, the association of hypoglycemia with mortality was cumulative to the associations of hyperglycemia and increased glycemic variability with mortality [20].

This investigation expands upon earlier work studying the association of hypoglycemia with mortality in the critically ill. It is notable that the investigators of the first prospective randomized controlled trial of IIT [4] and a subsequent confirmatory before and after investigation [5] stated that severe hypoglycemia (SH, defined as BG < 40 mg/dL) did not have an independent effect on mortality. However, a subsequent pooled analysis of the two Leuven trials [13] determined that the OR for mortality associated with a single episode of SH was 3.23 (2.25 to 4.64) (*P *< 0.0001). This compares to the OR for mortality associated with a single episode of SH reported from two large observational series, 2.28 (1.41 to 3.70) in a cohort of 5,365 mixed medical-surgical patients from a single ICU in the USA [10] and 2.6 (2.1 to 3.2) in a cohort of 66,184 patients admitted to 24 Australian ICUs [11]. Recently, Egi *et al. *investigated the relationship between milder degrees of hypoglycemia and mortality in a population of 4,946 patients admitted to two Australian ICUs with glycemic targets of 108 to 180 mg/dL [14]. Multivariate analysis revealed that patients with a minimum BG of 54 to 63 mg/dL had significantly higher risk of mortality than did those with a minimum BG of 72 to 81 mg/dL, OR 1.93 (1.27 to 2.95) (*P *= 0.002) - as well as significantly higher risk of infection - OR 2.16 (1.17 to 3.99) (*P *= 0.01). The findings of the current investigation corroborate the association of mild hypoglycemia with increased risk of mortality in critically ill patients demonstrated in the study by Egi and coworkers.

The strengths of the current investigation include, in part, the diverse nature of the different patient populations and the breadth of the dataset used for analysis. This international collaboration comprises large, well described observational cohorts from one ICU in the USA and three ICUs in The Netherlands, as well as the 21 ICUs participating in one of the major randomized controlled trials of intensive insulin therapy [8], increasing the generalizability of its findings. We acknowledge several limitations in the dataset used for this analysis. The use of bedside glucometers in a percentage of patients in all three cohorts and capillary fingerstick blood in a percentage of the patients in the ST and GL cohorts is a potential limitation as this technology has been associated with analytic inaccuracies in critically ill populations [21-23]. Nevertheless, this measurement technology reflects "real world" practices. Moreover, any "scatter" of the data induced by a measurement technology less accurate than a central laboratory analyzer would serve to "dampen" the signal of the association of hypoglycemia and mortality in this population. It is notable that Egi *et al. *[14], in a study that solely used arterial blood gas analyzers for glucose measurement, identified a threshold for the association between hypoglycemia and mortality that was similar to that seen in our investigation. While the manual and intermittent nature of monitoring BG in all three cohorts likely led to an incomplete accounting of all the hypoglycemia that occurred in the patients studied, due to the nursing time required for compliance with even hourly BG [24], it is likely that the size of the combined cohort was sufficient to examine the endpoints chosen. The absence of information about nutritional support, insulin treatment and glycemic control after ICU stay are additional limitations in the dataset used for this analysis. Finally, there are no data available to evaluate the relationship between the timing of the hypoglycemic events and death, or the specific causes of death for patients in the three cohorts.

There are some possible links between hypoglycemia and worsened outcome or complicated course of critical illness [25]. First, the physiological mechanisms triggered by hypoglycemia are commonly impaired during critical illness. These include the inhibition of insulin release, typically occurring when BG is lower than 80 mg/dl [26]; an increased release of glucagon, epinephrine and growth hormone when BG is lower than 65 mg/dl and increased release of cortisol when BG is lower than 55 mg/dl [27]. During critical illness, exogenous insulin is infused and the levels of glucagon, epinephrine, cortisol and growth hormone are typically already elevated. Second, large swings in BG, as observed when hypoglycemia is aggressively treated with a large amount of intravenous glucose, may be associated with cellular damages [28]. Unfortunately, data detailing the amount of glucose given to treat hypoglycemia and the neurological status of the patients in our three cohorts were not available. Third, the detrimental effects of hypoglycemia are well documented in the brain. Indeed, glucose is the preferential energetic substrate in the brain. The absence of cerebral stores of glucose and the diffusive character of transport imply that the glucose concentration in neurons and glial cells is entirely determined by BG [29]. Therefore, brain injured patients are at higher risk of hypoglycemia-related damage; conversely, hypoglycemia also induces brain dysfunction even in patients without prior cerebral compromise.

This study has a number of important clinical implications. While earlier investigations detailed the independent association of severe hypoglycemia with increased risk of mortality in critically ill patients [10-13], the data from the current work suggest strongly that milder degrees of hypoglycemia are also associated with harm. Moreover, mild hypoglycemia was associated with increased risk of mortality among patients admitted with medical as well as surgical diagnoses, among patients with diabetes and those without, and among patients with mild-moderate disease and moderate-severe disease, as reflected by APACHE II score (but not among patients with APACHE II scores ≥ 25, in whom the severity of illness may have overwhelmed the independent impact of hypoglycemia).

Interestingly, the signal for harm varied in the three cohorts. The relative risk of mortality associated with hypoglycemia was strongest for patients in the ST cohort, intermediate for patients in the GL cohort, and smallest for those in the NL cohort, inversely related to the prevalence of hypoglycemia in the three populations of patients. One explanation of this finding is the different distribution of severity of illness in the cohorts, as reflected by APACHE II scores. More patients in the NL cohort had APACHE II scores ≥ 25 than did patients in the ST and GL cohort. Figure 2 demonstrates that there was no association between hypoglycemia and mortality among patients in these groups; perhaps the severity of illness lowered the sensitivity of the association of hypoglycemia with increased risk of mortality. In addition, patients in the "strict" IIT cohort in the NL, with a glycemic target of euglycemia, were treated using a guideline that allowed brief excursions into the 60 to 80 mg/dL, resulting in a higher rate of hypoglycemia than was seen in the other cohorts. The IIT guideline used for the NL cohort also allowed patients to gradually recover from hypoglycemia, avoiding the administration of quantities of dextrose that might lead to sharp spikes in glucose levels and, consequently, excessive glucose variability, recently identified as having an independent association with increased risk of mortality in critically ill patients [13,30-32]. This practice could be a second reason that the sensitivity of the association between hypoglycemia and mortality was lowered in the NL-S cohort. Additional differences between the NL-L and NL-S cohorts included the exclusive use of arterial blood for measurement of BG in the IIT group, and significantly higher glycemic variability, as reflected by CV and SD, in the NL-S cohort. Thus, any future evaluations of the association of hypoglycemia with mortality in critically ill populations may benefit from an analysis of the severity of illness, the prevalence and practice of treating hypoglycemia, as well as the glycemic management protocol employed.

Our investigation contrasts with the finding in a recent study of a U-shaped curve relating mean BG to mortality in critically ill patients [33]. The lowest mortality was associated with a mean BG of 80 to 110 mg/dL and 110 to 140 mg/dL during ICU stay. Patients with hypoglycemia in these two bands of mean BG had very similar mortality, but 4.3 and 2.6 times the rate of mortality as those without hypoglycemia, respectively. Higher bands of mean BG were associated with even higher rates of mortality, both for patients with and without hypoglycemia. These observations are parallel to some of the key findings of the major interventional trials of intensive insulin therapy. The sharply higher mortality observed in patients with a mean BG of 80 to 110 mg/dL and hypoglycemia compared to those with a mean BG of 80 to 110 mg/dL and no hypoglycemia highlights the different outcomes of Leuven 1 [4] and 2 [6]. In the medical ICU trial [6] the markedly higher rate of severe hypoglycemia in patients attenuated the beneficial impact of intensive insulin therapy observed in the surgical ICU trial [4]. Moreover, while in both trials, the "conventional" arm had a glycemic target range of 180 to 200 mg/dL, our investigation demonstrated that patients with mean BG ≥ 180 mg/dL and no hypoglycemia sustained even higher mortality than did those with mean BG of 80 to 110 mg/dL with hypoglycemia. Finally, in our study, patients with hypoglycemia and a mean BG of 80 to 110 mg/dL sustained higher mortality than did those without hypoglycemia and a mean BG of 140 to 180 mg/dL, analogous to the conclusions of the NICE to SUGAR trial, in which the 13.6-fold higher rate of severe hypoglycemia in patients treated in the interventional arm compared to those in the control arm was associated with higher mortality [9].

## Conclusions

This investigation supports and extends the findings of other recent studies of the impact of hypoglycemia in the critically ill and suggests that mild hypoglycemia, defined as BG < 70 mg/dL, is independently associated with increased risk of mortality. Of course, it would certainly be unethical and, therefore, impossible to perform a randomized trial targeting hypoglycemia in a group of critically ill patients. However, while causality cannot be proven by these data - they must be considered hypothesis generating - the findings of this investigation suggest that critical care teams should attempt to avoid even modest degrees of hypoglycemia in their patients.

## Key messages

• Mild hypoglycemia, defined as BG < 70 mg/dL, was associated with increased risk of mortality in a diverse and heterogeneous group of three patient cohorts.

• The relative risk of mortality associated with hypoglycemia differed among the cohorts.

• The association of mild hypoglycemia with mortality was observed in medical and surgical patients, in diabetics and non to diabetics and it occurred independently of mean BG during ICU stay.

• The association of mild hypoglycemia with mortality was strongest in patients with mild to moderate and moderate to severe disease upon presentation to the ICU, reflected by APACHE II scores < 15 and 15 to 24, respectively, and was weaker in patients with APACHE II scores ≥ 25.

• Efforts to reduce the occurrence of mild hypoglycemia in critically ill patients may reduce mortality.

## Abbreviations

APACHE II: Acute Physiology and Chronic Health Evaluation II disease classification system; BG: blood glucose; CI: confidence interval; CV: coefficient of variation; DM: diabetes mellitus; GL: GLUCONTROL cohort; GL-C: GLUCONTROL-control arm; GL-C: patients in the control arm of the GLUCONTROL trial; GL-I: GLUCONTROL-intensive arm; GL-IIT: patients in the intervention arm of the GLUCONTROL trial; HYPO: hypoglycemia (BG < 70 mg/dL); LOS: length of stay; NL: Netherlands cohort; NL-L: Netherlands "loose" cohort; NL-S: Netherlands "strict" cohort; NON: no hypoglycemia; RR: relative risk; SD: standard deviation; ST: Stamford cohort.

## Competing interests

James S. Krinsley MD has performed consulting work for Medtronic Inc., Edwards Life Sciences, Baxter, Roche Diagnostics, and Optiscan Biomedical and has received speaker's fees from Edwards Life Sciences, Roche Diagnostics and Sanofi to Aventis. Marcus J. Schultz MD, PhD has performed consulting work for Medtronic Inc., Roche Diagnostics and Optiscan Biomedical, and has received research support from Optiscan Biomedical. Jean-Charles Preiser MD, PhD has performed consulting work for Medtronic Inc., Edwards Life Sciences and Optiscan Biomedical. All other authors have no competing interests.

## Authors' contributions

JSK wrote the initial and subsequent drafts of the manuscript and performed statistical analysis. MJS and JCP reviewed all drafts of the manuscript and assisted with revisions. PES, REH, FvBH, JPvdS and CM participated in data acquisition and reviewed the final draft of the manuscript. All authors have read and approved the final manuscript for publication.

## Supplementary Material

Additional file 1 **Stamford Hospital glycemic management protocol**. Click here for file

Additional file 2 **GLUCONTROL trial glycemic management protocols**. Click here for file
