# Supplementary material for: Mild hypoglycemia is independently associated with increased mortality in the critically ill
Source: Crit Care. 2011 Jul 25;15(4):R173. doi: 10.1186/cc10322 (PMC3387616; doi:10.1186/cc10322)
Supplement: Additional file 1 — Stamford Hospital glycemic management protocol. [file cc10322-S1.DOC]

SH ICU PROTOCOL FOR GLYCEMIC MANAGEMENT

Approved by the ICU Committee 1/9’07

# Rationale

Hyperglycemia is strongly associated with increased hospital mortality as well as organ system dysfunction among critically ill patients.

MD Signature____________________

Date_____________________

# Goal

The goal of this protocol is to maintain serum glucose **80-125 mg/dl**.

# Monitoring

Glucose levels will be evaluated ***by blood testing or fingerstick testing***, using the following schedules. For most patients, the 6AM glucose evaluation will be obtained from the morning BMP. If glucose 80-125 mg/dl for two consecutive days without treatment, glucose levels can be checked Q6 hours.

| Diet | **Frequency of monitoring** |
| --- | --- |
| NPO, tube feedings, TPN | Q3 hours |
| PO diet | 1 hour AC and QHS |

# **Treatment of hyperglycemia**

| **Glucose value** | Action |
| --- | --- |
| **<125** | No treatment |
| **125-149** | 4 units sc Aspart insulin; Recheck glucose value in 3 hours |
| **150-174** | 6 units sc Aspart insulin; Recheck glucose value in 3 hours |
| **175-199** | 8 units sc Aspart insulin; Recheck glucose value in 3 hours |
| **200-249** | 10 units sc Aspart insulin; Recheck glucose value in 3 hours |
| **250-299** | 12 units sc Aspart insulin; Recheck glucose value in 3 hours |
| **300+** | 14 units sc Aspart insulin; Recheck glucose value in 3 hours |

- If glucose value exceeds 180 on two successive measurements, start IV regular insulin infusion
- Hourly FSG or blood glucose measurements will be obtained in patients receiving insulin infusions.
- If glucose control stabilizes on the infusion, monitoring frequency can be decreased to Q2 hours.
- The above table is a guideline; it can be modified if the patient requires more or less intensive therapy.

Management of insulin infusion

## **Important points**

- All patients receiving continuous insulin must receive a continuous source of glucose, either via IV (D5W or TPN), or enteral feeds.
- The insulin infusion is discontinued if the patient has to leave the ICU for a diagnostic test as well as upon discharge from the ICU
- Variances to the protocol are documented by the RN in Meditech and countersigned by the house officer

Initial infusion rate: REGULAR insulin

| **Glucose value** | **Insulin dose** |
| --- | --- |
| **180-229** | 4 units/hour |
| **230-259** | 6 units/hour |
| **260-299** | 8 units/hour |
| **300-399** | 10 units/hour |
| **400+** | 12 units/hour |

**Management of glucose values < 100**

**Risk of hypoglycemia is increased:**

- **In patients with ESRD – *USE 50% dose***
- **In patients with severe liver failure**
- **When the rate of change in glucose levels is high after insulin treatment**

**Decrease the dose of insulin and increase the frequency of monitoring in these situations.**

| **<40** | Stop infusion, give 1 amp D50, check FSG in 1 hr |
| --- | --- |
| **40-59** | Stop infusion, give ½ amp D50, check FSG in 1 hr |
| **60-79** | Stop infusion, check FSG in 1 hr |
| **80-99** | Continue infusion at 1unit/hr |

**Transition from insulin drip:** Requires order from housestaff. For patients with stable insulin requirements on a drip, calculate the previous 24-hour total received, give 50% of this dose as Lantus insulin at midnight, and DC the continuous infusion at 6AM.

If this treatment plan does not lead to a decrease in the patient’s glucose values, contact the house officer.

Version 1.2 1/12/07
