# Supplementary material for: Mild hypoglycemia is independently associated with increased mortality in the critically ill
Source: Crit Care. 2011 Jul 25;15(4):R173. doi: 10.1186/cc10322 (PMC3387616; doi:10.1186/cc10322)
Supplement: Additional file 2 — GLUCONTROL trial glycemic management protocols. [file cc10322-S2.DOC]

**
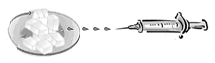
**

**GROUP IIT**

**GLUCONTROL**

**STUDY**

**TARGET BLOOD GLUCOSE LEVELS : 80 - 110 mg/dL**

1. Mix 1 Unit Regular Human Insulin per 1 cc 0.9% NaCl. Administer via infusion pump IV connected as close as possible to the patient (NB : the solution is stable only 24 hour after preparation).
2. Check blood glucose level with arterial blood sampling or capillary blood sampling *(blood gas analyser or Hemocue® device)*.
3. Check blood glucose level hourly until achievement of target value or when required by attending physician (eg after prescribing steroids,…).
4. During the four hours following achievement of target value (with a variation of blood glucose level < 50% of the two last measured values), check blood glucose level each two hours (if variation of blood glucose level is > 50%, check blood glucose level hourly). Then, for the next hours, with a variation of blood glucose level < 50% of the two last measured values, check blood glucose level once each four hour.
5. STARTING PERFUSION

| **Blood glucose level** | **Insulin infusion rate** |
| --- | --- |
| < 110 mg/dl | On hold |
| 110-140 mg/dl | 1 UI / H |
| 140-180 mg/dl | 2 UI / H |
| > 180 mg/dl | 4 UI / H |

NB : IV Bolus of 1-2 UI Insulin are allowed if target value is not fast enough reached.

1. MAINTENANCE PERFUSION

| **Blood glucose level** | **Incremental Insulin infusion rate** |
| --- | --- |
| >300mg/dl | +3 UI/H |
| 180-300mg/dl | +2 UI/H |
| 140-180mg/dl | +1 UI/H |
| 110-140mg/dl | +0,5 UI/H |
| 80-110mg/dl | + 0 UI/H (target range) |
| 40-80 mg/dl | Stop insulin,  Hourly control of glycaemia until >80mg/dl |
| <40 mg/dl | Stop insulin, 12gr glucose IVD,  Call immediately physician,  Hourly control of blood glucose level until >80mg/dl |

1. PARTICULAR CASES

- In case of prolonged discontinuation of nutritional support (e.g. : surgical procedure, CT Scanner, …), insulin perfusion will be interrupted (or may be adapted by the physician in charge of patient during the procedure.).
- After discharge of ICU, the intravenous infusion of insulin will be shifted to according to the standard local practice (subcutaneous administration, Oral AD, …).

**
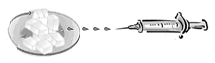
**

**GROUP LIT**

**GLUCONTROL**

**STUDY**

**TARGET BLOOD GLUCOSE LEVELS : 140 – 180 mg/dL**

1. Mix 1 Unit Regular Human Insulin per 1 cc 0.9% NaCl. Administer via infusion pump IV connected as close as possible to the patient (NB : the solution is stable only 24 hour after preparation).
2. Check blood glucose level with arterial blood sampling or capillary blood sampling *(blood gas analyser or Hemocue® device)*.
3. Check blood glucose level hourly until achievement of target value or when required by attending physician (eg after prescribing steroids,…).
4. During the four hours following achievement of target value (with a variation of blood glucose level < 50% of the two last measured values), check blood glucose level each two hours (if variation of blood glucose level is > 50%, check blood glucose level hourly). Then, for the next hours, with a variation of blood glucose level < 50% of the two last measured values, check blood glucose level once each four hour.
5. STARTING PERFUSION

| **Glycemia** | **Insulin infusion rate** |
| --- | --- |
| < 180 mg/dl | On hold |
| 180-250 mg/dl | 1 UI / H |
| 250-300 mg/dl | 2 UI / H |
| > 300 mg/dl | 4 UI / H |

NB : IV Bolus of 1-2 UI Insulin are allowed if target value is not fast enough reached.

1. MAINTENANCE PERFUSION

| **Glycemia** | **Incremental Insulin infusion rate** |
| --- | --- |
| >300mg/dl | +3 UI/H |
| 250-300mg/dl | +2 UI/H |
| 180-250mg/dl | +1 UI/H |
| 140-180mg/dl | + 0 UI/H (target range) |
| 80-140mg/dl | Stop insulin, check glycemia each 4 hour |
| 40-80 mg/dl | Stop insulin,  Hourly control of glycaemia until >80mg/dl |
| <40 mg/dl | Stop insulin, 12gr glucose IVD,  Call immediately physician,  Hourly control of glycemia until >80mg/dl |

1. PARTICULAR CASES

- In case of prolonged discontinuation of nutritional support (e.g. : surgical procedure, CT Scanner, …), insulin perfusion will be interrupted (or may be adapted by the physician in charge of patient during the procedure.).
- After discharge of ICU, the intravenous infusion of insulin will be shifted to according to the standard local practice (subcutaneous administration, Oral AD, …).
